# Skin diseases among elderly patients attending skin clinic at the Regional Dermatology Training Centre, Northern Tanzania: a cross-sectional study

**DOI:** 10.1186/s13104-016-1933-6

**Published:** 2016-02-22

**Authors:** Kelvin Mponda, John Masenga

**Affiliations:** Department of Dermatology, Queen Elizabeth Central Hospital, Blantyre, Malawi; Regional Dermatology Training Centre, Moshi, Tanzania; Kilimanjaro Christian Medical University College, Moshi, Tanzania

**Keywords:** Elderly, Skin diseases, Outpatient skin clinic, Tanzania

## Abstract

**Background:**

As global population of the elderly continues to rise, a critical need to provide it with health services, including dermatology, will be significant, especially in developing countries like Tanzania. To adequately meet their dermatologic needs, knowledge of local patterns of skin conditions is vital. This study was aimed to describe the spectrum of skin diseases among elderly patients attending skin clinic at the Regional Dermatology Training Centre (RDTC) in Northern Tanzania.

**Methods:**

A descriptive hospital based cross-sectional study was conducted between January 2013 and April 2013 at RDTC and included all patients aged 55 years and above who consented to be examined. Diagnoses were clinical, diagnostic tests being done only when necessary. Ethical clearance to conduct the study was granted.

**Results:**

A total of 142 patients, age ranges 55–99 years, median age of 67.5 years were seen. Eczemas were the leading disease group (43.7 %), with unclassified eczemas (33.9 %) predominating. Papulosquamous disorders (15.4 %) were second with psoriasis (50 %) being the leading disease. Infections (11.3 % with fungal infections the leading group representing 5.6 % of all diseases), tumours (9.8 %: Kaposi’s sarcoma 4.2 %), vascular disorders 9.1 % (lymphedema 4.9 %), autoimmune disorders 7.7 % (connective tissue diseases 4.9 %), vitiligo 4.2 %, nutritional diseases 2.1 % (pellagra 0.7 %), urticaria 0.7 % and drug reactions 0.7 %.

**Conclusions:**

Eczemas are the most common group of disorders among elderly patients presenting at RDTC.

## Background

As the steady rise in population of elderly people continues in both developing and developed countries, the impact of the need to provide special services including health to this growing population will be great [1–3]. In Tanzania, those aged 55 years and above are currently estimated to constitute 8.2 % of the total population, [4] up from 7.4 % in 8 years [5]. Such rise in the proportion of elderly people in different populations is regarded as the most important factor that will alter the prevalence and pattern of skin diseases in general dermatology practice [6]. Various age-related skin changes make skin diseases more frequent with advancing age [1, 7]. Despite health workers’ recognition that skin problems are ermomous [8] there is relative lack of population studies of skin diseases in medical scientific studies [9] including in dermatology, [10] and more so in the elderly population. [11] Only one study has been done in Africa looking at the pattern of skin diseases in the elderly, [12] and to our knowledge, no such study has been done in East Africa in general and in Tanzania in particular. If dermatologic needs of this population are to be adequately met by health practitioners, local data is vital since pattern of skin diseases in the elderly differs widely in various geographical settings [7]. The present study aimed to describe the pattern of skin problems affecting elderly patients seen at the Regional Dermatology Training Centre (RDTC).

## Methods

This was a cross sectional hospital based study carried out at the RDTC dermatology clinic in Northern Tanzania between January 2013 and April 2013. The RDTC was jointly established in 1992 by the Tanzanian Ministry of Health, the International Foundation for Dermatology and the Kilimanjaro Christian Medical Centre (KCMC) with an objective to improve prevention, treatment and rehabilitation of skin diseases, leprosy and sexually transmitted infections in Sub-Saharan Africa. Patients come from Tanzania and from neighboring countries. Data collection was done on normal clinic days where all patients are seen by dermatologists and dermatology residents under supervision of the dermatologists. Diagnoses were clinical, laboratory tests being done where necessary. Diseases were categorized as summarized in Table 2. A senior dermatology resident (KM) reviewed the files for the recorded diagnoses and for any relevant data, and did a full physical examination of the patients. A cut off age of 55 years was used to define old age for two reasons: Firstly, since there is no standard numerical criterion to define old age with, and that the United Nations’ (UN) cut off of 60 years might not adapt well to Africa, the UN recommends that a lower age cut off should be defined in populations like Africa [13]. While noting the impracticability of choosing a standard age for all populations to define old age with, the World Health Organisation (WHO) recommends 50 or 55 years of age in Africa for research purposes [13]. Secondly, whereas most nations use 65 years, the age at which people start receiving pensions as a cut-off, 55 years was chosen in this study by virtue of it being the optional retirement age in Tanzania [14]. Minimum sample size *n* was 100 calculated from formula *n* = [Z*ZP(1−P)]/(E*E), where P is percentage of elderly population attending the RDTC (7 % from clinic records), Z is z-score at 95 % confidence interval and E standard error at ±5 %. Data analysis was done using Statistical Package for the Social Sciences version 16 to describe frequencies.

Verbal informed consent was sought from all participating patients. Ethical clearance to conduct the study was granted by the Kilimanjaro Christian Medical College Research Ethics and Review Committee.

## Results

Out of 142 patients studied, 56.0 % were males. The median age of the study population was 67.5 years, ranging from 55 to 99 years. Sixty eight percent were married while 28.0 % were widowed. Most patients (51 %) had primary education, 29.0 % had gone beyond primary school while 21.0 % had no formal education. Diabetes mellitus was reported in 7 % and hypertension in 30 %.

The predominant complaint was itch (61 %), followed by ‘rash’ (56 %), pain (12 %), lump on their skin and dry skin (each 10 %), lower limb swelling and ulcer (each 4 %) and blisters (1 %). Other complaints (4 %) included pigmentary changes and their skin appearance being an embarrassment (Table 1). Of the 14 patients who complained of dry skin, 13 (93 %) also reported itch.

**Table 1:** Presenting complaints: frequency of the reported complaints

Presenting complaint	Frequency (%)
Rash	56
Pain	12
Swelling	4
Lump	10
Blistering	1
Dry skin	10
Ulcer	4
Itch	61
Other	4

Xerosis was the most commonly detected physical finding distributed as follows: upper limbs (50 %), trunk (48 %), generalized (46 %). Of note, all patients who complained of dry skin were also found to have xerosis on physical examination. The second most common physical finding was inter-digital maceration of the feet (39 %).

**Fig. 1:**
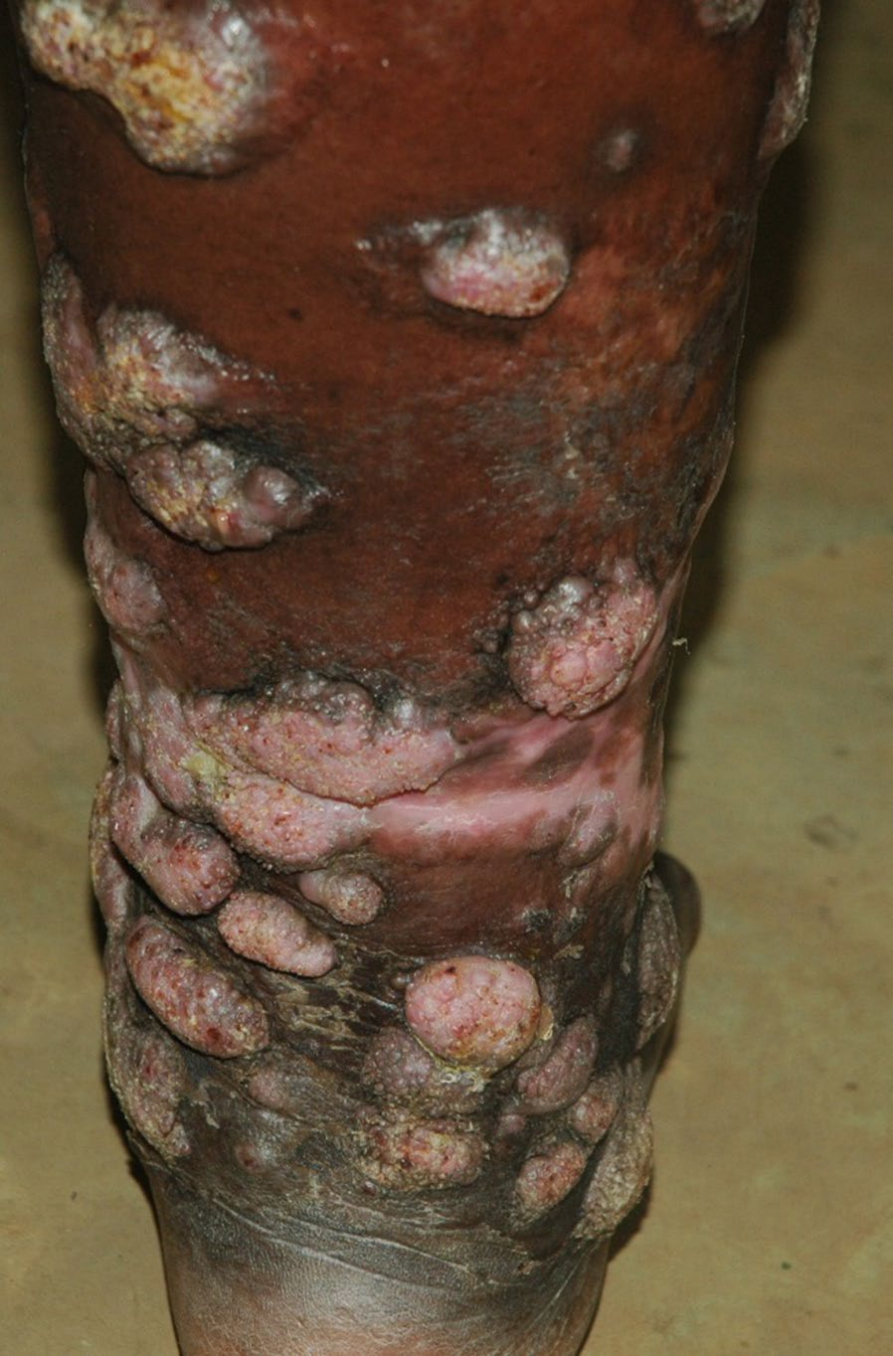
Chromoblastomycosis in a rural dwelling farmer

**Fig. 2:**
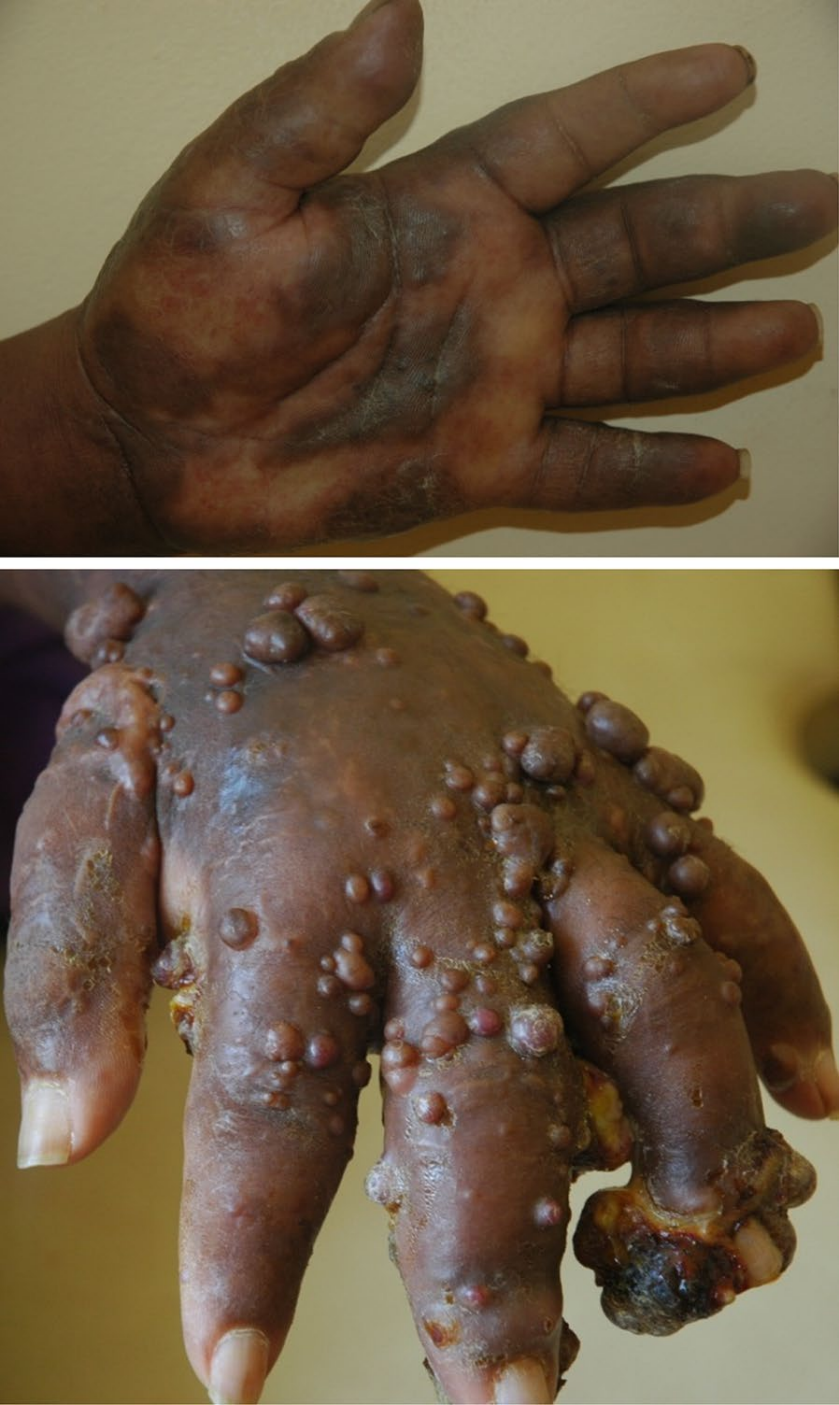
Kaposi sarcoma of the hand: African endemic (*above*), and Human Immunodeficiency Virus associated (*below*)

A wide spectrum of diseases was seen (Table 2). The group of eczematous disorders (43.7 %) was the leading disease group. The most frequent types of eczemas were: unclassified (where no further description had been provided) eczema (33.9 %), atopic eczema (21.0 %), xerotic eczema (17.7 %), stasis eczema (9.7 %), nummular eczema (8.1 %), seborrhoeic eczema (8.1 %) and contact eczema (1.6 %). The second most common group of disorders was of papulosquamous disorders (15.4 %) in which psoriasis (50.0 %), lichen planus (32.0 %) and keratodermas (18.0 %) were most frequent. The infections group made up 11.3 % of all diseases and was distributed as follows: fungal infections (50.0 %), bacterial infections (31.0 %) and viral infections (19.0 %). Fungal infections subgroup was distributed as follows: tinea pedis (50 %), pityriasis versicolor (12.5 %), onychomycosis (12.5 %), intertrigo (12.5 %) and chromoblastomycosis (12.5 %) (Fig. 1). Viral infections subgroup was distributed as follows: herpes zoster (67 %), herpes simplex (33 %). Bacterial infection subgroup was distributed as follows: impetigo and cellulitis (60.0 %) and leprosy (40.0 %). Tumours group of disorders (9.8 % of all diseases) included Kaposi’s sarcoma (KS) (43.0 %), keloids (29 %), mycosis fungoides (14.3 %), acral lentiginous melanoma (7.1 %), and basal cell carcinoma (BCC) (7.1 %). Of the KS cases, five were African endemic, while one was human immune deficiency virus associated (Fig. 2). The single case of BCC was in the only white patient in the study, originally from Europe but had lived in Tanzania for 15 years.

**Table 2:** Types of diseases

Disease category	Frequency	Percentage
Eczema	62	43.7
Unclassified eczema	21	14.8
Atopic dermatitis	13	9.2
Xerotic eczema	11	7.7
Stasis dermatitis	6	4.2
Seborrhoeic dermatitis	5	3.5
Nummular eczema	5	3.5
Contact dermatitis	1	0.7
Papulosquamous disorders	22	15.4
Psoriasis	11	7.7
Lichen Planus	7	4.9
Keratoderma	4	2.8
Infections	16	11.3
Fungal	8	5.6
Tinea pedis	4	2.8
Pityriasis versicolor	1	0.7
Chromoblastomycosis	1	0.7
Intertrigo	1	0.7
Onychomycosis	1	0.7
Viral	3	2.1
Herpes zoster	2	1.4
Herpes simplex	1	0.7
Bacterial	5	3.5
Superficial and deep	3	2.1
Leprosy	2	1.4
Tumours	14	9.8
Kaposi’s sarcoma	6	4.2
Keloids	4	2.8
Carcinomas	4	2.8
Vascular disorders	13	9.1
Lymphedema	7	4.9
Chronic venous insufficiency disorders	4	2.8
Hemangioma	1	0.7
Vasculitis	1	0.7
Autoimmune disorders	11	7.7
Connective tissue disorders	7	4.9
Blistering diseases	4	2.8
Vitiligo	6	4.2
Nutritional and systemic diseases	3	2.1
Pellagra	1	0.7
Sarcoidosis	1	0.7
Diabetic ulcers	1	0.7
Urticaria	1	0.7
Drug reactions	1	0.7

## Discussion

The prevalence of itch was higher than that commonly reported in literature [15] and in previous similar studies [16, 17]. This could be explained by the high prevalence of eczemas and papulosquamous disorders in our study. ‘Rash’ literary translated *‘*upele*’* in local Swahili language was reported in close to 2/3 in our study. The term *‘*upele*’* however is non-specific and may refer to any unusual spot on the skin surface. Its wide meaning may be responsible for the high prevalence of rash in our study compared to 15.6 % in the United States of America [16]. Xerosis was detected in over half of the study patients of whom only 1/10 gave a history of dry skin compared with 85.1 and 60.6 % respectively found in another study [16] in which 89.7 % were self reported or known to have ‘major medical illnesses’. Such medical problems can render elderly people less mobile. Immobilized status in geriatric populations has been reported to contribute to significant decrease in the general stratum corneum water content, [18] Only 7 and 30.1 % were diabetic and hypertensive respectively in this study even though we did not assess their morbidity.

As found in previous similar studies in which their frequency ranged from 11.9 to 58.7 %, [12, 19–21] eczemas were the leading group of disorders in this study. The prevalence of papulosquamous disorders also compares with 4 and 17 % found in previous studies. [21, 22] Global prevalence of psoriasis is estimated at 1–2 % [23] Other studies have revealed psoriasis prevalence ranging from 2.2 to 3.9 % [16, 19–21] which are fewer compared to 7.7 % in this study. Many patients with chronic diseases with significant psychological impact like psoriasis are referred from within and outside the country to RDTC, which is the only centre in Tanzania and, probably in East Africa, with a well established and relatively well-equipped dermatology unit. For the majority of such patients, treatment by phototherapy with psoralen or a dermatologist’s consultation can only be accessed at the RDTC. The higher prevalence of lichen planus in our study compared to that found in other studies (0.3 and 1.5 %) [17, 21] could be due to different physical and environmental factors in these settings, not assessed in our study, which have been reported to affect prevalence of this disease [24].

We found generally lower prevalence of infections than those found in Taiwan and in India (43.5 and 58.9 % ) [20, 22]. Fungal infections (5.6 %), viral infections (4.2 %) and bacterial infections (3.5 %) were all found to be lower compared to 16.9, 6.8 and 8.7 % respectively found by Souissi et al. in outpatient clinics in Tunisia [12]. Under reporting to our clinic might be due to financial constraints or, more importantly, appropriate treatment by general practitioners in primary and secondary health facilities. To our knowledge, the observed prevalence of KS has never been reported in any previous similar studies. This might be a reflection of higher seroprevalence of Kaposi sarcoma herpes virus (KSHV) infection in our population compared to other parts of world since KS is known to result from KSHV infection [25]. Local incidence of classic and endemic KS in particular reflect seroprevalence of KSHV in a particular geographic setting and is known to be high in sub-Saharan and Mediterranean countries [26]. Prevalence of the other remaining tumours is accounted for by almost all of our study population being of black race. The predominance of keloids in our study compared with 1 % in Taiwan is not surprising since keloids are known to be more prevalent in patients of African descent [27]. The more pigmented skin in Africans is protective from ultraviolet (UV) induced malignant skin cancers which explains their lower rates in our study than in Canada [17] and in Taiwan [20]. BCC and squamous cell carcinoma occurrences are reported to be inversely proportional to ‘degree of skin pigmentation in the population’ as the larger, more melanized melanosomes in the epidermis of dark skin filter twice as much ultraviolet B radiation as does that in Caucasians, providing an inherent sun protection factor of 13.4 in blacks [28]. Apart from the single case of BCC, the rest of the cancers seen were those in which exposure to UV rays is not a directly important risk factor.

The major limitation of the study is the small sample size and its hospital setting which does not allow generalizing but nevertheless offers an informative overview of the burden of skin diseases in elderly patients at this referral clinic.

## Conclusions

Eczemas are the predominant group of skin diseases among elderly patients presenting at RDTC, most of whom report itch. The dermatologic needs of this growing population will have to be met by Tanzanian dermatologists for many years to come.

## Abbreviations

RDTC: Regional Dermatology Training Centre
KCMC: Kilimanjaro Christian Medical Centre
KS: Kaposi sarcoma
KSHV: Kaposi sarcoma herpes virus
BCC: basal cell carcinoma
UV: ultraviolet
